# A Presumed Sickle Cell Anemia Crisis Revealed To Be Medium Vessel Vasculitis

**DOI:** 10.7759/cureus.36993

**Published:** 2023-04-01

**Authors:** Arwa Badeeb

**Affiliations:** 1 Radiology, King Abdulaziz University, Faculty of Medicine, Jeddah, SAU

**Keywords:** vaso-occlusive crisis, vasculopathy, medium vessel vasculitis, abdominal crisis, sickle cell anemia

## Abstract

A 24-year-old male sickle cell anemia patient presented with acute abdominal pain. Computed tomography (CT) demonstrated signs of bowel ischemia about the terminal ilium. He underwent bowel resection and anastomosis. Pathology of the resected bowel showed acute inflammation at the site of bowel perforation. This was thought to be secondary to bowel infarction from sickle cell vasculopathy. Despite the surgical intervention, the patient’s symptoms continued to worsen. He also developed bilateral toe pain during the same hospital stay. Reviewing the patient’s CT lower extremity runoff revealed no vascular thrombosis but rather medium vessel changes. The intra-abdominal arterial branches and the lower extremity vessels showed intermittent areas of vascular narrowing, wall thickening, and associated micro-aneurysms mainly of the distal hepatic arterial branches. These vascular changes posed a diagnostic dilemma as it is inconsistent with sickle cell anemia, which is known to cause vascular angiopathy as the underlying etiology of vaso-occlusive crisis. The literature lacked reports of any specific intra-abdominal vascular findings by imaging in sickle cell anemia. With the continued worsening of the patient’s condition, vasculitis was considered as an alternative diagnosis. The patient was empirically treated with steroids after which his symptoms improved. Unfortunately, he passed away after developing a large intracranial hematoma days after the initiation of steroid therapy. This report highlights the diagnostic dilemma of vaso-occlusive crisis versus vasculitis in sickle cell anemia patients.

## Introduction

Abdominal pain is a common symptom in sickle cell anemia (SCA) patients. The differential diagnosis in these cases encompasses gallstone disease, splenic sequestration crisis, and vaso-occlusive crisis causing bowel ischemic changes [1-3].

The literature does not disclose any vascular changes in the medium vessels in the abdomen associated with SCA [4]. However, in brain imaging of SCA patients, it has been described that they may present with infarcts secondary to abnormal vascular changes related to Myomoya syndrome [5].

This case report presents a patient with SCA who was initially misdiagnosed with abdominal vaso-occlusive crisis as a cause of bowel perforation and, hence, his abdominal complaints. Eventually, he was treated with steroids for vasculitis, which resulted in symptomatic improvement. This case highlights a diagnostic dilemma, as the literature lacks a description of the abdominal vascular changes related to SCA. Furthermore, there have been no reports on the association between vasculitis and SCA. To the best of our knowledge, this report is the first to highlight such diagnostic challenges in the radiology literature.

## Case presentation

This is the case of a 24-year-old male who was diagnosed with sickle cell anemia. He presented to a community hospital with chest and acute abdominal pain. Initial computed tomography (CT) showed acute pulmonary emboli, left pneumothorax, and free air in the abdomen. Because the patient required a tertiary center level of care, he was transferred to our institution. On admission, the patient was already intubated with vital signs indicating shock. His abdomen was distended with absent bowel sounds. There were absent breath sounds on the left chest as well. According to his family, this was the patient’s first hospital admission. He had no prior surgeries and was not on any medical therapy.

Laboratory investigations showed an elevated white blood cell count (WBC) of 23.27, low hemoglobin (Hb) level of 7.4, normal platelet count, elevated international normalization ratio (INR) of 2.2, low protein S activity of 66.3, low protein C activity of 53, elevated Factor VIII assay of 267.4, normal anti-thrombin III, and normal lupus anticoagulant.

**Figure 1:**
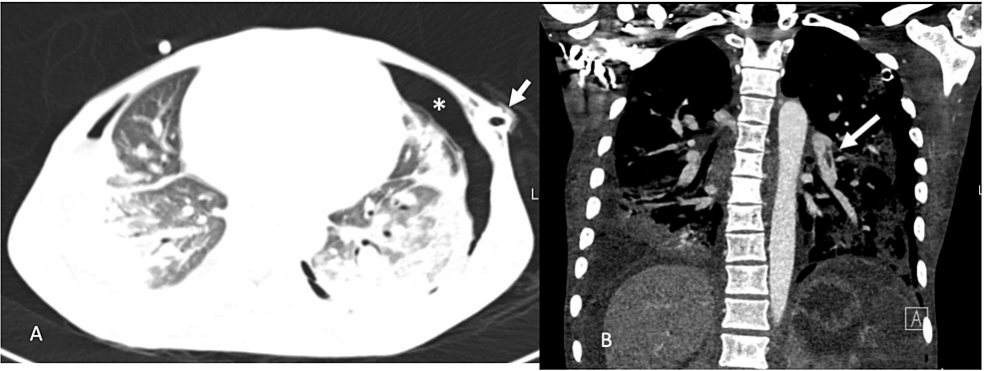
CT pulmonary angiogram of the patient at the time of presentation. (A) Bilateral pneumothoraxes indicated by the (*) on the left. Chest tubes were placed on presentation (white arrow points at the left-sided chest tube). (B) Central left-sided lobar pulmonary arterial filling defect indicated by the white arrow consistent with acute pulmonary embolism.

**Figure 2:**
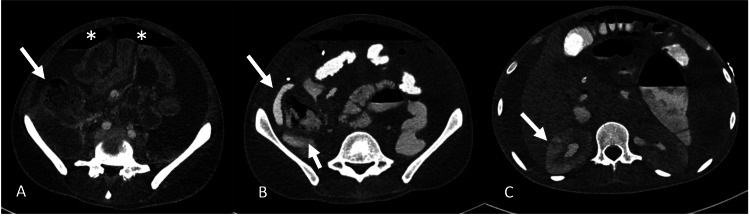
CT abdomen and pelvis at the time of the presentation. (A) Initial late arterial phase sequence without oral contrast showing sluggish to non-enhancement of the cecum (white arrow) with free intraperitoneal air as indicated by (*). (B, C) Repeat scans performed on the same day but a few hours later with oral and no intravenous contrast; (B) Oral contrast extravasation adjacent to the cecum indicating perforation; (C) Delayed contrast excretion from segments of the kidney indicating acute renal impairment.

CT chest (pulmonary embolism protocol) and abdominal and pelvic CT (in the porto-venous phase) were performed at presentation to the emergency department. Chest CT showed acute central pulmonary emboli, airspace opacities at the periphery of both lungs, and left-sided pneumothorax with a mediastinal shift to the right (Figure 1). The abdomen revealed diffuse hypoattenuation of the spleen, indicating infarction, wedge-shaped areas of renal hypoenhancement mainly in the right kidney, hypoenhancement of the ileal loops at the terminal ilium, and foci of free intraperitoneal air concerning bowel perforation secondary to infarction (Figure 2).

Initially, the working diagnosis was sickle cell crisis with a hypercoagulable state. The patient was then treated accordingly. A left chest tube was placed to relieve the pneumothorax, and heparin was infused to treat the pulmonary emboli. The patient was also rushed to the operating room (OR) for a possible infarcted bowel and perforation. The OR notes mention ischemic changes and a focal area of perforation at the terminal ilium, for which the infarcted segment was resected with a right lower quadrant ileostomy. Histopathological examination of the resected terminal ileum revealed extensive serositis.

The patient was transferred to the intensive care unit (ICU) and was treated for severe sickle cell crisis with end-organ damage, including splenic, renal, and bowel infarctions. The patient remained in the ICU for almost two months, with no significant improvement in his overall health.

**Figure 3:**
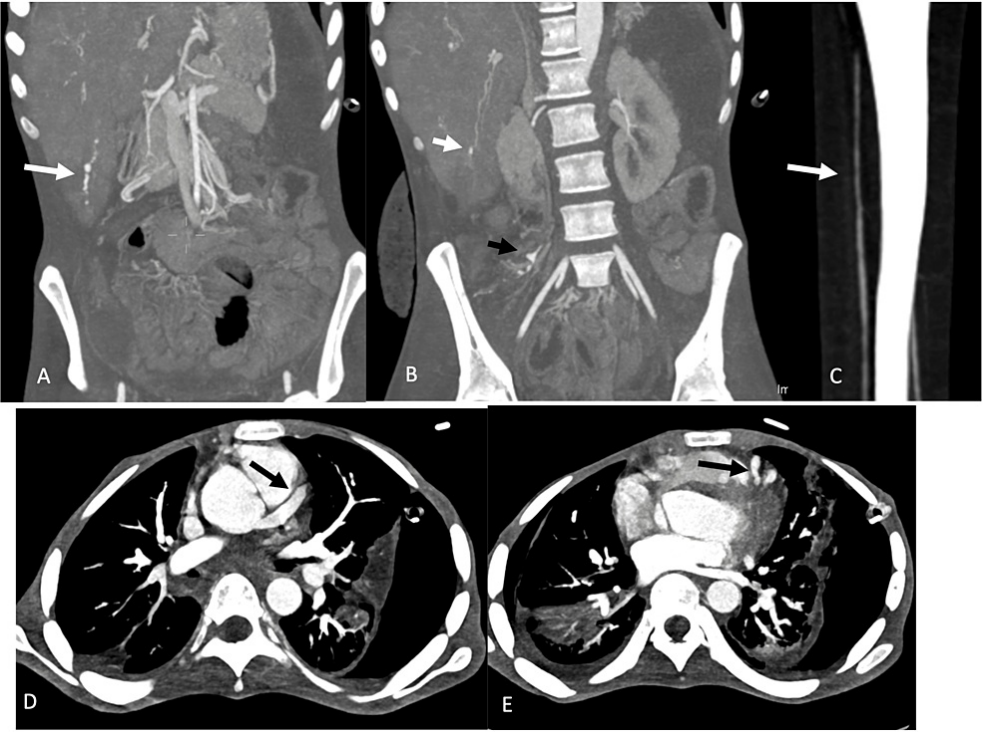
CT angiogram of the chest, abdomen, and lower extremity runoffs follow-up after two months; performed as the patient complained of toe pain. (A, B) Coronal maximum intensity projection (MIP) images of the abdomen showing abnormal beaded appearance of the right hepatic artery with micro-aneurysms (white arrows) and similar appearance to the ileocolic artery (black arrow). (C) Coronal MIP image of the right leg showing subtle arterial luminal irregularity of the right anterior tibial artery. This is less apparent than the visceral arteries in A and B. (D, E) Axial MIP images of the chest showing saccular dilatation of the proximal left anterior descending artery (D) and distal areas of beaded appearance (E).

During his hospital stay, he developed bilateral toe pain and signs of ischemia on examination, which required a CT angiogram (CTA) of the lower extremities. CTA did not reveal any acute occluding clots that required surgical intervention but rather unexpected findings of an abnormally beaded appearance of some of the intraabdominal medium-sized arteries, mainly the right renal artery, superior mesenteric artery (SMA), and its distal branches, especially the distal ilio-colic branches that lead to infarction of the bowel loop that was resected. Similar changes were observed in the splenic artery. Microaneurysms were observed in the distal right and left hepatic arteries. One of the previous CTA chest follow-ups also revealed ectatic changes in the coronary arteries (Figure 3).

The constellation of these changes raised concerns about medium-vessel vasculitis rather than sickle cell crisis. Rheumatology consultation and laboratory tests were performed. The management team empirically initiated the patient on a course of intravenous steroids. Results for the antinuclear antibody (ANA), antineutrophil cytoplasmic autoantibody, cytoplasmic (C-ANCA), perinuclear antineutrophil cytoplasmic autoantibody (P-ANCA), Serum anti-proteinase 3 antineutrophil cytoplasmic autoantibody (ANCA PR3), and myeloperoxidase antineutrophil cytoplasmic antibody (ANCA MPO) returned negative results.

The patient demonstrated excellent symptomatic improvement over the subsequent days. However, shortly after, he died from a large intracranial hemorrhage.

## Discussion

The World Health Organization (WHO) had estimated that 7% of the world’s population carries a hemoglobinopathy gene and 50% of these carry the SCA gene. In endemic regions such as the African continent and the Mediterranean region (including Arab countries), the prevalence of SCA can reach 20/1,000 [6].

SCA is a hereditary multi-system disease, and patients frequently present to hospitals with a spectrum of clinical presentations. Acute painful crisis is the most frequent presentation. Up to 50% of patients will present to a hospital before the age of five years [7]. Abdominal crisis, although infrequent, is one of the major issues. In general, abdominal crisis encompasses a range of underlying etiologies such as gallbladder disease, splenic sequestration, and vaso-occlusive crisis involving the bowel [2], with rare occurrences of bowel ischemia and infarction [8-10].

Recurrent sickling of red blood cells containing hemoglobin S (HbS) causes hemolysis or microvasculature occlusion. Additionally, SCA patients enter a hypercoagulable state due to the chronic depletion of nitrous oxide, which is a potent inhibitor of platelet activation. Chronic nitrous oxide depletion results in vasoconstriction and proliferative vasculopathy. Activation of procoagulant factors in the blood causes further aggregation of sickled blood cells and intraluminal thrombosis [4,11]. This process eventually results in vascular occlusion and tissue ischemia with end-organ damage causing pain. SCA crises usually have no obvious underlying causes. However, they can be attributed to infection, dehydration, stress, hypoxia, and other factors [4,8]. The patient in this report presented to our institute in an advanced severely ill condition where he was already intubated. Moreover, the initial presentation and nature of the inciting factors remain unclear to clinicians.

Patients with vaso-occlusive crisis generally present with varying levels of severity [4]. Ultrasound of the abdomen is usually ordered to exclude biliary disease; however, CT of the abdomen and pelvis is required in severe abdominal crisis to exclude bowel ischemia, as in our patient [1].

Our patient presented with an unstable case of bowel ischemia/infarction with perforation and end-organ damage, including the spleen and kidneys. In addition, the patient was in a hypercoagulable state with acute pulmonary emboli. All these manifestations were explained by a sickle cell crisis. It is found that 75% of SCA abdominal crises are related to gastrointestinal vaso-occlusive ischemia [3]. However, abdominal CT findings in our patient revealed a beaded appearance of medium vessels and small microaneurysms. There is no evidence in the literature that associates these features with SCA abdominal crisis. In general, there are no extra-cranial medium vessel changes reported in the radiology literature to associate with any of the sickle cell crises.

In contrast, the arterial vascular radiologic changes in the brain of SCA patients are confirmed and described in detail in the literature; this is referred to as Moyamoya syndrome. Sickle cell vasculopathy is the hallmark of many neurological manifestations. Twenty-five percent of patients will present with neurological manifestations and up to 55% will show vascular abnormalities by MRI [12]. These radiologic brain vascular changes include areas of marked arterial vascular narrowing intermittently with areas of dilatation and abnormal tufts of telangiectatic vessels. Histopathological studies show segmental arterial wall thickening with total luminal occlusion secondary to intimal hyperplasia, atrophy, and fibrosis of the media added to luminal thrombosis [5,11-13]. This brain involvement in SCA is a major concern as patients can present with significant neurological deficits, in the form of infarcts, seizures, and hemorrhages. The same pathological mechanism and histopathological changes are also attributable to the development of pulmonary hypertension, priapism, and leg ulcers in SCA patients [11,12,14].

However, these medium vessel arterial vascular changes have not been reported in the abdomen of SCA patients; the literature lacks pathological and radiological reports to disclose these changes. The only pathological changes described in the literature attributed to vaso-occlusive crisis in SCA are post-capillary venule occlusions, such as small vessel vasculitis, low flow state, and veno-occlusive disease [8]. Thus, post-capillary microvascular occlusion explains the patchy segmental changes in the bowel, mesenteric engorgement, and lack of arterial thrombosis [1-3].

A single case was reported in the literature in 1987, in which an autopsy of a patient with SCA demonstrated systemic necrotizing vasculitis [15]. Unfortunately, since autopsies are not performed in our institute, the exact pathology that led to the patient’s death remains unclear.

The CT findings of the patient in the current report feature a beaded appearance of medium vessels in the abdomen, pelvis, and lower extremities, along with mild aneurysmal dilatation of the coronaries and numerous microaneurysms of the hepatic arteries. These findings are usually reported in patients with medium-vessel vasculitis, especially polyarteritis nodosa (PAN). PAN is a segmental transmural necrotizing vasculitis that affects small and medium visceral arteries. This would weaken the vessel wall and result in aneurysm formation. PAN is usually not associated with ANCAs [4].

With the limited feasibility to perform vessel biopsies, CTA or angiography is usually appropriate for the initial diagnostic imaging workup of vasculitis. Angiography has fallen out of favor due to its invasive technique. Additionally, CT can assess vascular wall enhancement and provides information on end-organ damage. However, due to the limited spatial resolution of imaging, it is usually used for large and medium vessel vasculitis. Small vessel changes are not usually seen. Other imaging modalities including positron emission tomography (PET) imaging, MRI, and ultrasonography may have additive roles if further workup for large vessel vasculitis is needed but there is no strong evidence of their use in medium vessel vasculitis [16].

The patient in this case report demonstrated radiologic and laboratory results consistent with medium-vessel vasculitis. Moreover, the improvement in the patient’s symptoms supports the diagnosis of vasculitis rather than SCA abdominal crisis. The delay in diagnosis and treatment of the patient probably led to mortality.

## Conclusions

The obvious provisional diagnosis of abdominal complaints in SCA cases is sickle cell crisis. However, other differential diagnoses should be included at the beginning. The lack of documented arteriovascular changes in the abdomen of patients with SCA poses a diagnostic dilemma. This raises the need to further evaluate the intraabdominal vasculature in SCA by imaging and histopathology to create a baseline reference to the documented findings in the literature and hence avoid misdiagnosing vasculitis for sickle cell crisis in the future.
